# Overcoming heuristics that hinder people’s acceptance of climate-change-mitigation technologies

**DOI:** 10.3389/fpsyg.2025.1433280

**Published:** 2025-06-18

**Authors:** Anke Blöbaum, Karolin Schmidt, Michael Böcher, Julia Arlinghaus, Friederike Krause, Ellen Matthies

**Affiliations:** ^1^Faculty of Natural Sciences, Institute of Psychology, Environmental Psychology, Otto-von-Guericke-University Magdeburg, Madgeburg, Germany; ^2^Faculty of Humanities, Chair of Political Science and Sustainable Development, Institute for Social Sciences, Otto von Guericke University Magdeburg, Madgeburg, Germany; ^3^Institute for Engineering of Products and Systems, Department of Production Systems and Automation, Otto von Guericke University Magdeburg, Magdeburg, Germany; ^4^Inter 3—Institute for Resource Management, Berlin, Germany

**Keywords:** climate protection, climate change mitigation technologies, intervention evaluation, restriction heuristic, vignette methodology

## Abstract

The overall research objective of the present study is the investigation of the effects of a strongly expressed restriction-oriented climate change mitigation heuristic (SER heuristic) on people’s attitude toward and acceptance of climate change mitigation technologies such as Carbon Capture and Utilization (CCU). Furthermore, we want to examine the effects of a scenario-based communication intervention approach on the promotion of a supportive attitude toward and acceptance of CCU, especially referring to people characterized by a SER heuristic. Against this background, we present empirical findings based on an online experiment including a scenario-based intervention in an initial sample of 401 German participants. In line with our expectations, our findings show that participants characterized by a SER heuristic report a significantly lower supportive attitude toward CCU as well as a lower acceptance of CCU, compared to participants who are not characterized by a SER heuristic. Furthermore, our findings imply the examined scenario-based communication intervention approach to be an effective tool for the promotion of participants’ supportive attitude toward CCU and acceptance of CCU. Taken together, the present study provides further valuable insights for the promotion of people’s supportive attitude toward as well as of their acceptance of necessary new climate change mitigation technologies such as CCU.

## Graphical abstract

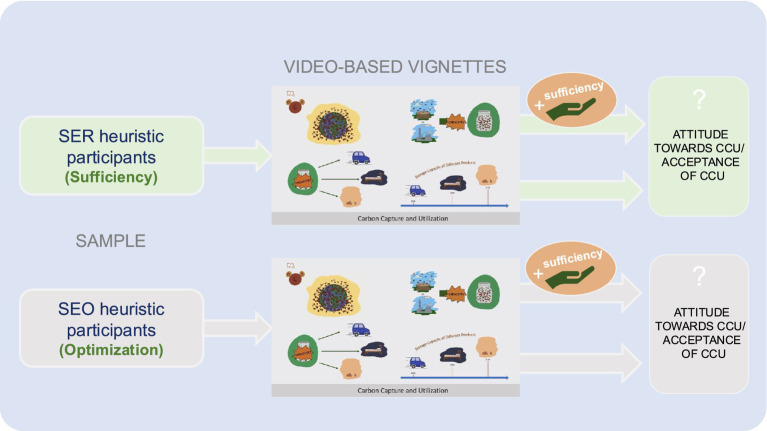

## 1 Introduction

In order to keep global warming below 2°C, as specified in the Paris Agreement, global green house gas (GHG) emissions must be reduced by 27% from 2019 levels by 2030. The Intergovernmental Panel on Climate Change (IPCC) has made it clear, that although this target is still achievable in principle, it requires immediate and effective action, including technological measures such as the creation of carbon sinks (IPCC, 2022). These measures require not only the development of innovative technologies, for example for Carbon Capture and Storage (CCS) or Carbon Capture and Utilization (CCU) (IPCC, 2022, 2023; Napp et al., 2017), but also the social acceptance of these technologies for their effective use. The implementation of measures to comply with the international climate agreement requires a transformation of our society not only toward more sufficiency, but rather toward a net-zero society (IPCC, 2018), in which the remaining, unavoidable anthropogenic emissions are counteracted by anthropogenic decomposition (storage in carbon sinks). Even if demand is drastically reduced (e.g., by reducing consumption and promoting the circular economy), products—including carbon-neutral product alternatives—must still be manufactured, albeit in smaller quantities (Perišić et al., 2022; Stegmann et al., 2020). Bioeconomic solutions are therefore considered relevant, as they promise to substitute fossil resources with renewable raw materials in industrial and energy production, aiming to shift economic production toward a bio-based economy (Böcher et al., 2020). The urgently needed transformation of our society must therefore be seen as both a technical and a societal challenge. The societal challenge in this respect relates not only to strengthening sufficient lifestyles, but also to the acceptance of particularly high-impact, innovative technologies (e.g., CCS and CCU).

### 1.1 Relevance of climate change mitigation technologies

Against the backdrop of an envisaged transformation toward a net-zero society, great hope lies in the potential of climate change mitigation technologies (CCMTs). There are already many well-established technologies that are helping to mitigate climate change. Some directly reduce carbon emissions by shifting to renewable energy sources such as wind turbines and solar power. Others contribute indirectly by enhancing energy and resource efficient processes, such as through genetic engineering. In this context, alternative production methods are also being investigated that indirectly reduce GHG emissions such as methane, e.g., lab-grown meat or insect-based protein (Tuomisto et al., 2022). Additionally, some technologies focus on climate mitigation by defossilizing production processes (e.g., biorefineries) while others directly aim to reduce global warming (e.g., solar radiation management). To compensate non-avoidable or difficult-to-avoid emissions CCMTs that bind GHGs through physical, chemical and biological processes and store them in the long-term are considered to be highly important (see Hoyos and Tait, 2025 for an overview) and are widely discussed in terms of climate policy in the European Union (EU) and Germany (Schenuit et al., 2023).

CCU and CCS are at the heart of current solutions for capturing as much CO_2_ as possible from fossil-based processes (Sunaryo et al., 2023). The CO_2_ captured in the process can either be stored (storage) or used directly as a carbon source (utilization) to prevent the CO_2_ from reaching the atmosphere and contributing to climate change. CCU goes beyond pure sequestration and aims to convert the captured carbon into products, thus creating a circular carbon economy (Leung et al., 2014).

Particularly among some environmental organizations and activists, CCMT are discussed controversially. CCS in particular is discussed very critically here, while CCU is viewed rather positively overall. A joint press memo of Greenpeace Germany, Friends of the Earth Germany (BUND) and others (Greenpeace, 2024) criticizes CCS as a bogus solution inter alia by preventing the phase-out of fossil fuels and the shift toward a circular economy. On the other hand, other German environmental protection organizations, such as NABU and WWF, have recently changed their minds about carbon capture and now consider CCMTs as necessary complimentary solutions for climate protection (BDI et al., 2024). This may be due to the differentiating attitude of environmental associations in the course of concepts of ecological modernization (Mol, 2000).

### 1.2 Acceptance of climate change mitigation technologies

The basis for the acceptance of CCMTs within the population of the EU—and thus of people from countries that have a significant impact on global climate change—appears to be sound [European Commission, 2023; Bundesministerium für Umwelt, Naturschutz, nukleare Sicherheit und Verbraucherschutz (BMUV) and Umweltbundesamt (UBA), 2023]. In the Euro-Barometer, 77% of European citizens agreed that “climate change is a very serious problem” (European Commission, 2023). There also appears to be a high level of willingness to mitigate climate change through individual behavior. While this is encouraging in the fight against the climate crisis, it seems that individuals see their role in climate protection primarily in changing individual lifestyles and focusing on less impactful curtailment behaviors (Bouman et al., 2020; Jakučionytė-Skodienė and Liobikienė, 2021; Whitmarsh, 2009).

Environmental psychology research already provides a series of empirical findings on psychological and situational predictors of individual pro-environmental behaviors, in particular, curtailment behaviors: We know about the impact of norms, values and beliefs on behaviors such as private energy consumption, choice of transport, or food consumption (Bamberg et al., 2007; Fritsche et al., 2018; Klöckner, 2013; Steg et al., 2014; Stern, 2000; Thøgersen, 2006). Following Stern’s conception (Stern, 2000) we understand curtailment behavior and the support of climate protection measures (here CCMTs) as *different types* of pro-environmental behavior. The Value-Belief-Norm (VBN) theory explicitly considers these different types of environmentally friendly behavior (Stern, 2000) and assumes that these behaviors are preceded by an activated personal norm, a moral obligation to behave in an environmentally friendly manner (Schwartz and Howard, 1981). This normative activation, in turn, is preceded by a causal chain of various factors, beginning with biospheric, altruistic and egoistic values (De Groot and Steg, 2008). In particular, the importance of altruistic and biospheric values has been successfully demonstrated for a variety of CCM behaviors, both for restrictive behaviors (Whitley et al., 2018) and for technology acceptance (Bidwell, 2023). These values, which are at the origin of the chain of effects, do not appear to directly influence behavior. Instead, their impact is mediated through a set of cognitive and emotional variables. At the highest level of mediation between values and individual problem awareness are convictions, which essentially consist of assumptions about economic growth and the relationship between humans and nature. Based on the conception and findings of Matthies et al. (2023), we will refer to these beliefs as “*heuristics*.”

Given the urgency of mitigating climate change, research should not focus primarily on supporting curtailment behaviors (Nielsen et al., 2024). But while we know about climate change awareness and willingness to contribute to climate change mitigation through individual lifestyle changes (see above), empirical research on the support for CCMTs is not as advanced and provides ambivalent results (see Baum et al., 2024). More research on the acceptance of these technologies and—given that they are innovative and still relatively unknown—the effectiveness of impact-related knowledge transfer is needed in this context.

It is by no means certain that information about the potential of these technologies will lead to greater acceptance among all groups of people who are seriously concerned about climate change, nor that existing motivations to adopt a sufficiency-oriented lifestyle will simply be supplemented by additional support for innovative CCMTs. This overlooks, among other factors, the embedding of different behaviors in their respective socio-spatial context and the associated behavioral constraints. However, we see great potential in examining beliefs (i.e., heuristics) that are important for the process of norm activation.

The results of Matthies et al. (2023) even tend to indicate that the intention to realize a sufficient lifestyle as well as the acceptance of CCMT might be based on very different heuristics, which can also hinder each other (see 2.3).

### 1.3 Climate change mitigation heuristics: restriction versus optimization?

Heuristics have been addressed as a research topic in various disciplines for 50 years, and the definitions are correspondingly diverse and sometimes vague (Hjeij and Vilks, 2023). In psychology, heuristics are defined as cognitive processes that enable people to make a quick decision by consciously or unconsciously ignoring parts of the available information (Gigerenzer and Gaissmaier, 2011; Korteling et al., 2023). This becomes particularly relevant in more complex decision-making situations. Kahneman (2011) defines heuristics as “a simple procedure that helps find adequate, though often imperfect, answers to difficult questions” (Kahneman, 2011, p. 98).

Based on Simon’s (1955) research on bounded rationality, Gigerenzer developed the concept of positive ecological rationality. Instead of dealing with the tendency to fail or the claimed irrationality of human thinking in complex situations, he was interested in the human ability to select heuristics like a tool from an adaptive toolbox in order to solve problems quickly and intelligently in a complex world characterized by uncertainties (Gigerenzer, 2008). A heuristic is considered ecologically rational if it is best adapted to the context or surrounding ecosystem (Gigerenzer, 2015). The tools from this adaptive toolbox, i.e., the heuristics, are not limited to assumptions, methods and rules, but can also include social rules and consistent beliefs that help to simplify and accelerate decision-making processes in complex situations.

When it comes to pro-environmental behavior and decision-making in this context, we are also dealing with complex situations: there are non-linear, linear and noisy relationships between inputs and outputs, decisions are interrelated and can have ambiguous and cumulative outcomes, and our environment can change as a result of the decisions we make, but may also change autonomously (Osman, 2010).

Accordingly, heuristics have also been shown to be relevant in the context of pro-environmental behavior, e.g., when choosing food based on its environmental impact (Wassmann et al., 2023) or when processing green advertising (Santa and Drews, 2023).

Following this understanding and in line with Matthies et al. (2023), we understand climate-change-mitigation heuristics as assumptions or consistent beliefs that guide human judgments about the appropriateness of climate-friendly behavior.

With regard to previous psychological research in the field of selective exposure, such climate-change-mitigation heuristics are likely to play an important role in how people perceive, process, and evaluate information. According to the classic assumption of selective exposure theory, individuals tend to defend their beliefs against potential challenges by engaging in selective exposure (see, e.g., Festinger, 1957). Selective exposure refers to the tendency to avoid information that contradicts one’s beliefs and instead seek out information that supports them. This cognitive bias has been referred to as congeniality bias (e.g., Eagly and Chaiken, 1998) or confirmation bias (e.g., Jonas et al., 2001). In their meta-analysis, Hart et al. (2009) provided empirical evidence not only for a general preference for congenial over uncongenial information, but also for moderating factors—most notably, the value-relevance of the beliefs in question. Specifically, the congeniality bias tends to more strongly influence selective exposure when the belief is highly relevant to an individual’s personal values.

Against this background, it becomes clear that people’s climate-change-mitigation heuristics, as described below, may significantly affect their judgments regarding the appropriateness of climate-friendly behavior.

#### 1.3.1 Restriction heuristic to mitigate climate change

The study by Matthies et al. (2023) demonstrated that a heuristic, which is closely linked to the tradition of the environmental protection movement and was essential in driving forward the commitment to nature conservation and climate protection, is now affecting the acceptance of high-impact climate protection mitigation technologies: the restriction heuristic. This heuristic is rooted in concerns about excessive consumption and limited resources.

The publication of the Club of Rome’s report ‘The Limits to Growth’ (Meadows et al., 1972) launched a controversial debate on the role of Western economic systems and their narrative of unlimited growth as the main cause of the environmental crisis. This important discourse gave impetus to the environmental movement of the 1970s, which advocated for sustainable development and a more sufficient way of life, thus challenging the prevailing global hegemony of the growth paradigm of the 1950s and 1960s (Schmelzer, 2017). The narrative or heuristic of frugality and restriction gained momentum particularly in response to the oil crises of the 1970s, that exposed the vulnerabilities of fossil fuel-dependent economies and the increasing scarcity of resources. The environmental movements responded by promoting practices such as recycling, energy conservation, and sustainable living as alternatives to the prevailing culture of consumerism and overconsumption. This sufficiency or restriction heuristic was further developed in the late 1980s and, in addition to considerations of sufficiency, also included questions of a fair global distribution of development opportunities (Brundtland, 1987) and discussions on consequences of carbon emissions. In line with the global equity perspective, the budget approach, particularly in the context of the Kyoto Protocol, revolves around the concept of allocating carbon emission ‘budgets’ to different countries based on their historical contributions to greenhouse gas emissions and their respective mitigation capabilities. In essence, the budget approach seeks to distribute the burden of emission reductions fairly, taking into account historical emissions and the capacity of countries to adapt to and mitigate climate change (Messner et al., 2010). Matthies et al. (2023) assume that a generalized heuristic of restriction has emerged from this discourse, which also integrates the moral obligation to restrain, as a kind of compensation for historical overconsumption (Barclay et al., 2005). Within the present study, we will examine effects of a strongly expressed restriction-oriented climate change mitigation heuristic (hereinafter referred to as SER heuristic). According to the above-mentioned considerations, we assume a SER heuristic to be determined by dominant views on the implementation of climate protection measures, which have to be associated with a morally conditioned restriction for individuals. In contrast, climate protection measures that are not characterized by such strong restrictions in individual lifestyles (like CCMTs), are consequently considered as less important despite a high impact for climate protection.

Although this restriction heuristic is effective in limiting and compensating for individual overconsumption, the latest the report of IPCC (2022) shows that sufficiency measures alone will not be adequate to mitigate climate change; economic instruments and new technologies are urgently needed. In this context, however, the results of Matthies et al. (2023) show that a dominant (very strong) restriction heuristic can become an obstacle to people’s acceptance of newer and highly effective technologies.

#### 1.3.2 Optimization heuristic to mitigate climate change

Matthies et al. (2023) contrast the heuristic of pure restriction and refraining with a heuristic that focuses on high-impact behaviors (optimization heuristic). The results indicated that this optimization heuristic not only supports an openness to effective and new behaviors to mitigate climate change, but also proves to be the more relevant heuristic for climate change mitigation behavior in general. This optimization heuristic is characterized by an active search for solutions and an openness to different as well as technology-related strategies.

In the present study, we also examine effects of a strongly expressed optimization-oriented climate change mitigation heuristic (hereinafter referred to as SEO heuristic). We assume that the SEO heuristic is primarily characterized by a focus on climate protection measures that are perceived as highly effective (such as CCMTs), whereas measures that entail significant restrictions on individual lifestyles are regarded as less important. This heuristic does not represent a counterproposal that focuses exclusively on technical solutions, but rather an extended heuristic that is aimed at effective climate protection strategies and technologies without refraining from sufficiency strategies.

This raises the question of how individuals with a very dominant restriction heuristic might be able to shift toward an optimization heuristic.

The keen problem awareness among people with a dominant restriction heuristic suggests that they have considerable potential to engage in climate protection measures, which makes them particularly interesting. What might hinder the transformation of the restriction heuristic in the direction of an optimization heuristic? What concerns or fears could this be based on?

The resistance against technological solutions may be related to the history of the environmental movement itself (see above), i.e., to the experience of the various generations of the environmental movement that the narrative of sufficiency could only be introduced into the social discourse characterized by the growth paradigm with great effort (Schmelzer, 2017). Strategies to promote innovative CCMTs such as CCU or CCS may inadvertently raise fears that they are primarily aimed at maintaining current living standards without seriously tackling climate change. This perception risks undermining the full potential of CCMTs, as the technical solutions are perceived as merely avoiding efforts. If the resistance is indeed based on the fact that technological measures and investments are associated with the risk that sufficiency strategies should be avoided, i.e., are interpreted as an avoidance strategy, then a communication strategy that provides information about the impact relevance of these technologies while guided by appreciative communication regarding the necessity of sufficiency strategies, should be able to increase the acceptance of CCMT.

### 1.4 Research questions and research aims

The overall research objective of the present study is the investigation of the effects of a strongly expressed restriction-oriented climate change mitigation heuristic (SER heuristic) on individuals’ attitude toward and acceptance of CCMTs such as CCU in contrast to individuals characterized by a strongly expressed optimization-oriented climate change mitigation heuristic (hereinafter referred to as SEO heuristic). We chose CCU because it is an innovative and highly effective technology which, at the time of data collection, was not as controversial as CCS in terms of potential risks (see 2.1 for details). Furthermore, we wanted to examine the effects of a scenario-based intervention approach on individuals’ attitude toward and acceptance of CCU, especially referring to individuals characterized by a SER heuristic. The core aspect of this intervention is to not only provide information about the climate mitigation potential of this technology, but also to convey the necessity of sufficiency strategies in an appreciative manner in order to prevent mistrust that technological strategies are being used to avoid sufficiency measures.

With regard to the initial findings from Matthies et al. (2023), the following (baseline-) hypotheses were tested:^1^

H0a: Participants characterized by a SER heuristic (hereinafter referred to as SER heuristic individuals) report significantly lower supportive attitude toward CCU than participants characterized by a SEO heuristic (hereinafter referred to as SEO heuristic individuals).

H0b: SER heuristic individuals report significantly lower acceptance of CCU than SEO heuristic individuals.

We further formulated several research hypotheses with regards to the overall effects of a scenario-based intervention approach on SER heuristic individuals’ attitude and acceptance exclusively. By doing so, we examined the effects of two different scenario types. On the one hand, we investigated effects of a scenario in which the combination of various climate protection strategies (i.e., sufficiency, efficiency, and the use of CCMTs such as CCU) leads to the achievement of national climate protection goals (hereinafter referred to as “baseline vignette”). On the other hand, we also examined the effects of a slightly modified scenario-based intervention, which included an additional appreciative emphasis on the importance of the sufficiency strategy (hereinafter referred to as “supplementary vignette”).

We tested the following hypotheses referring to both scenario-types:

H1a: Presenting the baseline vignette significantly increases SER heuristic individuals’ supportive attitude toward CCU.

H1b: Presenting the baseline vignette significantly increases SER heuristic individuals’ acceptance of CCU.

H2a: Presenting the supplementary vignette leads to a significantly greater increase in the SER heuristic individuals’ supportive attitude toward CCU compared to the presentation of the baseline vignette.

H2b: Presenting the supplementary vignette leads to a significantly greater increase in the SER heuristic individuals’ acceptance of CCU compared to the presentation of the baseline vignette.

Finally, we also examined the possible effects of the examined scenario-based intervention approach on SEO heuristic-individual’s attitude and acceptance of CCU. Since we expected SEO heuristic individuals to be basically characterized by relatively high supportive attidues toward CCU as well as by high acceptance of CCU (as described above, see H0a and H0b), we believe, there should therefore be no relevant scope or need for change in both dependent variables for SEO heuristic individuals at all. That is why, the following hypothesis was tested:

H3: Referring exclusively to SEO heuristic individuals, neither the baseline vignette, nor the supplementary vignette leads to significant changes in individuals’ attitude toward CCU or acceptance of CCU.

All hypotheses and analyses were pre-registered unless stated otherwise.

## 2 Materials and methods

### 2.1 Participants and procedure

Data were assessed via an online survey developed with the SoSci-survey software.

At the beginning of the survey, participants were inquired on the overall inclusion-criteria for the study. We used the inclusion-criteria procedure to make sure that only people with a fundamentally clear tendency in their assessment of different sustainability strategies (SER vs. SEO heuristics) took part in the actual survey at all (see inclusion criterion procedure for details).

In order to make sure that all participants were familiar with CCU, the inclusion-criteria procedure was followed by the presentation of a short video providing general information about CCU (see Appendix B.1 for details on the information provided in the video). The video-presentation was followed by the pre-measurement of the dependent variables (see below for details) and the measurement of participants’ expression of a restriction vs. optimization heuristic, which we used as the central criterion for group formation (SER heuristic- vs. SEO heuristic individuals) in the data analysis.

Afterwards, participants were randomly assigned to the two different vignette-based intervention-conditions and received a video-based vignette intervention (baseline vignette vs. supplementary vignette). Finally, the post-measurement of the dependent variables was conducted, and the survey closed by measuring participants’ sociodemographic features.

#### 2.1.1 Creating a scenario-based intervention approach

The intervention was realized by experimental vignette methodology (EVM). EVM attempts to present the participants with a scenario that is as realistic as possible. The participants should put themselves in the situation presented in order to make a decision under the given contextual conditions (Maddux and Rogers, 1983). Vignettes have already been applied to different scientific fields after being introduced by Rossi et al. (1974).

Vignettes are not designed to create a realistic image of reality (Wallander, 2012) or to induce immersion, because the focus is not on whether the generated scenario corresponds to reality, but rather on whether the activation of thought and behavioral processes is comparable to real life (Schmidt et al., 2022). Some studies have indeed shown that participants behave similarly in hypothetical and real scenarios and make comparable decisions (e.g., Peabody et al., 2004; Shah et al., 2007; Veloski et al., 2005). Following Atzmüller and Steiner (2010), we developed the vignettes considering experimental aspects (which are mainly manipulated), controlled aspects and additional contextual aspects to enrich the scenario without affecting the dependent variable.

**Figure 1:**
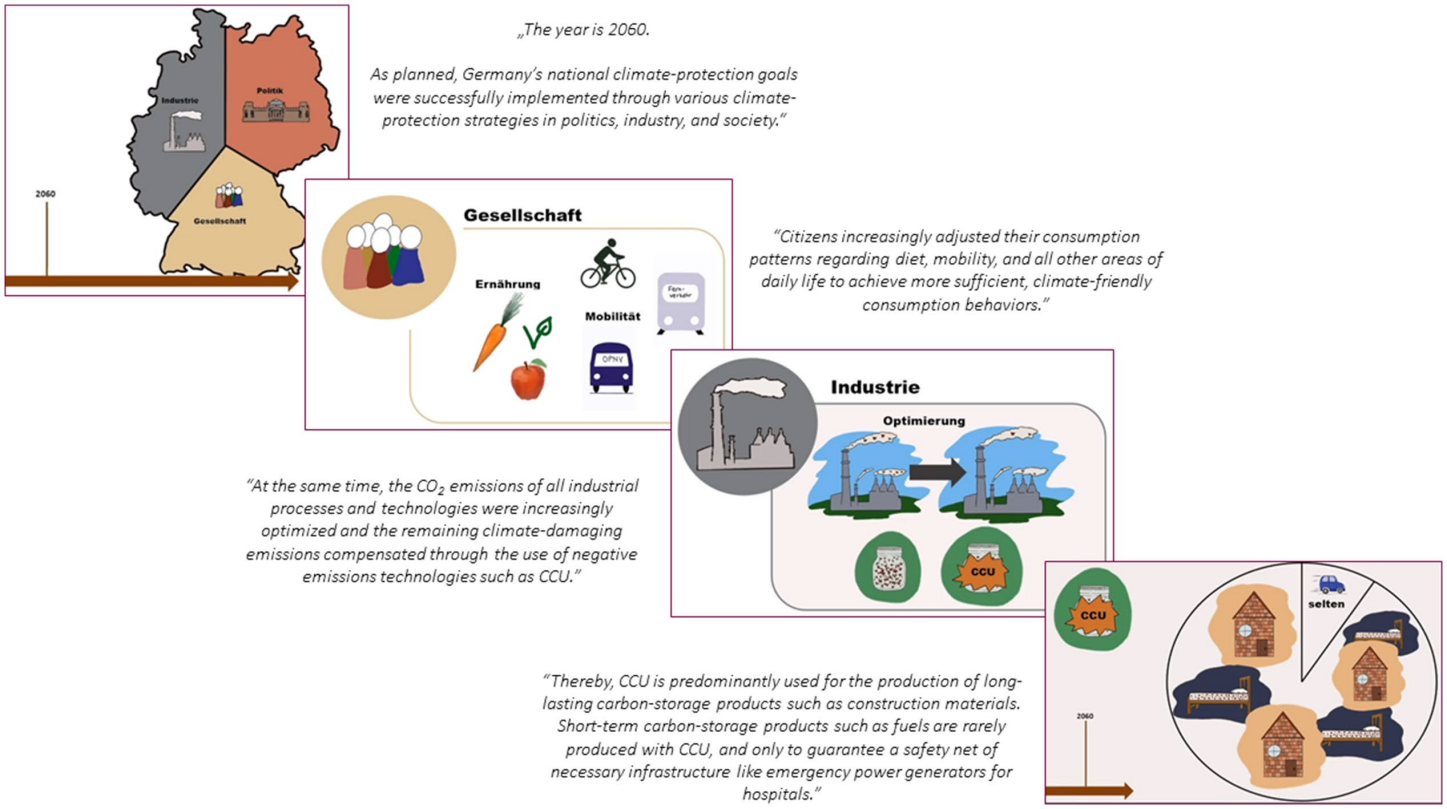
Baseline vignette video.

**Figure 2:**
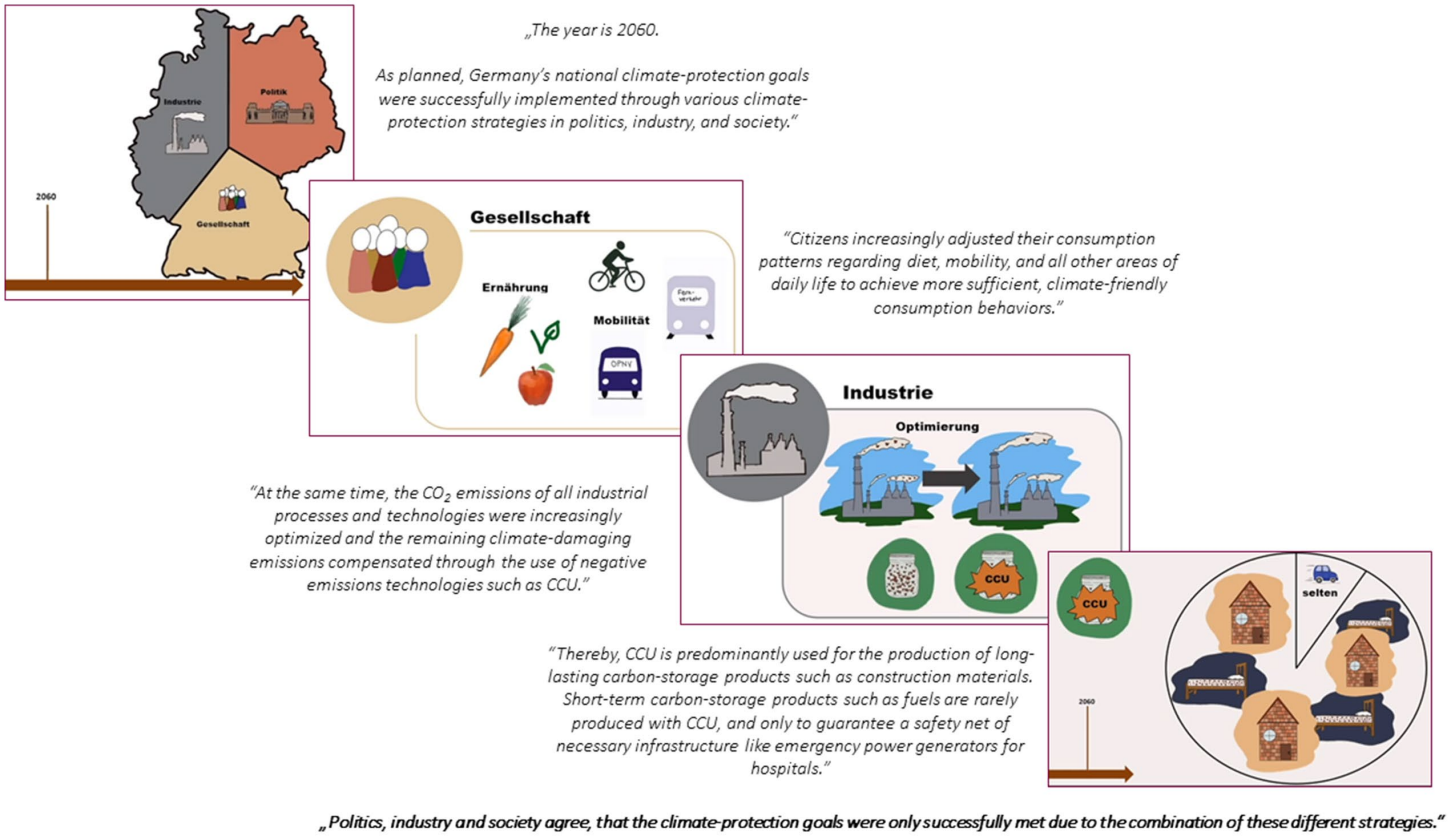
Supplementary vignette video.

Two different video-based vignettes were realized. Vignette 1 (baseline vignette) depicted a future scenario in which the combination of different climate protection strategies (sufficiency and efficiency strategies as well as the use of technologies such as CCU) leads to the fulfillment of national climate protection targets. The baseline vignette video lasted 1 min and 10 s. The information was presented in German and was acoustically conveyed, accompanied by suitable visual elements such as images and symbolic illustrations (see Figure 1 for the illustrations used and Appendix B.2 for details on the information presented in the baseline vignette video). In addition to the information provided in the baseline vignette, vignette 2 (supplementary vignette) also included an explicit appreciation of the importance of sufficiency strategies at the end of the video. The supplementary vignette video lasted 1 min and 34 s. The information was also presented in German and, as with the baseline video, was acoustically conveyed and supported visually with appropriate images and symbolic illustrations (see Figure 2 for the illustrations used and Appendix B.3 for details on the information presented in the supplementary vignette video).

#### 2.1.2 Inclusion criterion procedure

As mentioned above, the survey started with an inclusion criterion procedure in order to make sure that only individuals with a fundamentally clear tendency in their assessment of different sustainability strategies (SER vs. SEO heuristics) took part in the survey at all. This procedure was highly important, since our study was explicitly focused on the examination of individuals characterized by a strongly expressed restriction heuristic (SER heuristic individuals). Therefore, it was necessary to directly exclude all individuals from participating in the survey who generally did not want to deal with the meaning of the two strategies in more detail and/or did not express a clear preference for one the two strategies.

Within the inclusion criterion procedure, we initially presented a short introduction-text on two different strategies for the achievement of national climate-protection goals in Germany (SER vs. SEO heuristics) to make sure that all participants were familiar with the general features of both climate-protection strategies:

*“Please read the following information text on the implementation of Germany’s national climate protection targets at your leisure and then answer the corresponding question:*

*Germany’s national climate protection targets can be achieved through various types of climate protection measures. Sufficiency measures effectively reduce greenhouse gas emissions by enabling citizens to increasingly align their consumption patterns in terms of food, mobility and all other areas of daily life with climate-friendly consumption. Efficiency measures, on the other hand, reduce greenhouse gas emissions through technical solutions, for example by increasing the energy efficiency of technical devices and industrial production processes and/or replacing fossil fuels with renewable energies.”*

The presentation of this short introduction-text was then followed by the inclusion criterion-question: *“In your opinion, which of the two climate protection measures presented (sufficiency measures or efficiency measures) is more important for the long-term achievement of Germany’s national climate protection targets? Please think carefully about which of the two measures seems more important to you and try to decide in favor of one of the two measures.”* When answering the inclusion criterion-question, participants’ could choose between the following options: “sufficiency measures,” “efficiency measures,” “unfortunately, I cannot decide—both types of measures are equally important” and “no answer.” Only participants, who chose the “sufficiency measures”—or the “efficiency measures”-option were then forwarded to the actual survey.^2^

#### 2.1.3 Exclusion criterion and final sample

Data for the present study were collected across Germany from 13 to 27/09/2023 via an online survey. We followed the APA guidelines on the ethical conduct of research. According to German law, the present study did not require ethical approval for our survey as anonymity of the data was ensured, no sensitive data were assessed, and no experimental interventions were carried out. Participant acquisition and data collection was carried out by the panel provider company Bilendi, which ensured that all participants are at least 18 years old and German residents.

As we aimed to examine a special target group in this study (in particular individuals characterized by a strongly expressed restriction heuristic (SER heuristic individuals), as well as individuals characterized by a strongly expressed optimization heuristic (SEO heuristic individuals) in comparison), a comparatively comprehensive procedure to exclude unsuitable participants was necessary to form the final sample.

Of the 2,720 persons who visited the link sent out by the panel provider company, a total of 1804 answered the questions implemented in the inclusion criterion procedure (see above) at the beginning of our survey. 81 persons were excluded from the survey, since they chose the “no answer”-option in the inclusion criterion procedure, while 642 persons had to be excluded due to their “unfortunately, I cannot decide—both types of measures are equally important”-answer.

Following the inclusion criterion procedure, *N* = 1,081 participants were forwarded to the survey. Out of these, we excluded all participants who did not complete the whole survey and/ or gave a wrong answer to an attention check question referring to the CCU-information video (see Appendix B for details), resulting in a sample size of *N* = 418. After deleting all cases which spent too little time on the whole survey (i.e., less time spent than the mean time—1.5× standard deviations), the sample was reduced to *N* = 401.

Deviating from the pre-registration, we then implemented the final exclusion procedure by considering participants’ values in the holistic^3^ measure representing participants’ expressed restriction heuristic (see Section 3.2 for details). The holistic measure could be calculated for *N* = 392 participants (M = 0.11, SD = 0.83, Min = −3.00, Max = 3.00). Since our study was focused on individuals who are characterized by a strongly/expressed restriction/ optimization heuristic (not by a medium expressed restriction/ optimization heuristic), participants with values in the medium range of our holistic measure (i.e., values greater than or equal to −0.50 and smaller or equal 0.50) were finally excluded from the sample. Based on this exclusion-criterion, our final sample was formed with *N* = 176 (with N_SER heuristic_ = 100 and N_SEO heuristic_ = 76). Table 1 gives an overview on the sociodemographic features of the final sample.

**Table 1:** Sociodemographic features of the sample (*N* = 176).

Sociodemographic feature	Sample
Gender	
Male	60.8%
Female	38.6%
Diverse	0.6%
Age	
M (SD)	47.62 (14.33)
Min	19
Max	69
Education	
Primary school not completed	1.1%
Primary school completed	6.3%
Secondary education	23.9%
Higher education entrance qualification	29.6%
University degree	38.6%
Household income per month	
Less than € 500	4.0%
€ 500–less than € 1,000	5.1%
€ 1,000–less than € 1,250	4.5%
€ 1,250–less than € 1,500	6.3%
€ 1,500–less than € 2,000	5.7%
€ 2,000–less than € 2,500	13.1%
€ 2,500–less than € 3,000	14.2%
€ 3,000–less than € 3,500	6.3%
€ 3,500–less than € 4,000	10.8%
€ 4,000–less than € 5,000	17.6%
More than € 5,000	12.5%

### 2.2 Measures

#### 2.2.1 Dependent variables: supportive attitude toward CCU and acceptance of CCU

The measurement of participants’ supportive attitude toward CCU as well as their acceptance of CCU in the present study was generally based on scales/items which were already used in preliminary studies (Matthies et al., 2023). By doing so and with regard to previous research on social acceptance of sustainable and renewable energy technologies (see, e.g., Bonaiuto et al., 2024 for an overview), on the one hand, we considered items that clearly referred to the concept of “acceptability,” representing a favorable orientation, i.e., the attitude toward CCU. On the other hand, we used items with a stronger focus on intended positive behavioral responses toward CCU and, thus, items that refer more to the oncept of “acceptance” (see Section 5 for further considerations on these concepts). In order to identify the most appropriate item-scale-structure based on the captured data in the present study, we finally conducted an exploratory factor analysis with all items considered for the measurement of the dependent variables (see Table A.2 in Appendix A for an overview of the results of the factor analysis). Based on these conceptual considerations as well as on the identified factorial structure of the present data, we finally measured participants’ attitude toward CCU, by asking them “*What is your attitude toward Carbon Capture and Utilization (CCU; CO_2_ capture and subsequent use of carbon*, e.g.*, bioplastics as a building material) as a technology to limit climate change?*” with answering-options ranging from (1) “very much against it” to (5) “very much in favor.” Furthermore, we asked for participants’ agreement referring to the following statement “*How much do you agree with the following statements about Carbon Capture and Utilization [.] as a technology to limit climate change? I support Carbon Capture and Utilization*.” With answering-options ranging from (1) “strongly disagree” to (5) “strongly agree.” Referring to both questions, participants’ answers were measured referring to CCU in general, as well as specifically referring to the use of CCU for the medium-term storage of emissions (from the production of plastics) and for the long-term storage of emissions (from the production of building materials). Thus, altogether, we used 6 items to measure participants’ attitude toward CCU, aggregated into an overall attitude-scale characterized by very high reliability in the pre- (α = 0.92) as well as in the post-measurement (α = 0.92).

To measure participants’ acceptance of CCU, we asked for their agreement referring to the following statement “*How much do you agree with the following statement about Carbon Capture and Utilization [.] as a technology to limit climate change? I try to convince others of the importance of Carbon Capture and Utilization*” with answering-options on a Likert scale ranging from (1) “strongly disagree” to (5) “strongly agree.” Again, we measured participants’ agreement to this statement referring to CCU in general, as well as specifically referring to the use of CCU for the medium-term storage of emissions (from the production of plastics) and for the long-term storage of emissions (from the production of building materials). Against this background, we integrated three items into an overall acceptance-scale characterized by very high reliability in the pre- (α = 0.93) as well as in the post-measurement (α = 0.92).

#### 2.2.2 Independent variables: expressed restriction heuristic and expressed optimization heuristic

In order to measure participants’ strongly expressed restriction/ optimization heuristic, we used scales/ items from prior research: Analogous to the measurement procedure from Matthies et al. (2023), we measured participants’ expressed restriction heuristic with a scale consisting of five items (e.g., “*How much do you agree with the following statement? We have asked far too much of our planet in recent years, so now we have to pay the price and do without*.”; (1) “strongly disagree” to (5) “strongly agree”), characterized by very high reliability (α = 0.94).

Furthermore, we measured participants’ expressed optimization heuristic with a scale consisting of four items (e.g., “*How much do you agree with the following statement? As citizens of an industrialized nation, we can contribute to solving the global climate crisis primarily through investment.*”; (1) “strongly disagree” to (5) “strongly agree”), which showed acceptable reliability (α = 0.73).

Within data analysis and in line with Matthies et al. (2023), we calculated a difference-value for each participant from both scales (restriction heuristic—optimization heuristic), representing a holistic measure for participants’ expressed restriction heuristic (with positive values representing a more dominant restriction heuristic, while negative values represented a more dominant optimization heuristic).

## 3 Results

Statistical analyses were computed with the Statistical Package for the IBM SPSS Statistics 28 (IBM Corporation, 2022).

### 3.1 Differences in attitude toward and acceptance of CCU between SER vs. SEO heuristic participants

We examined possible differences in the dependent variables (pre-measurement) between SER and SEO heuristic participants by computing a MANOVA with both dependent variables (attitude toward CCU and acceptance of CCU) and group assignment (SER vs. SEO heuristic participants) as the independent variable. The data analysis revealed a significant overall effect [*F*_(2, 146)_ = 12.632, *p* < 0.001; η_p_^2^ = 0.15]. As summarized in Table 2, SER heuristic participants (attitude: M_SER_ = 3.74, SD_SER_ = 1.02; acceptance: M_SER_ = 2.59, SD_SER_ = 1.29) were characterized by lower values on both dependent variables than SEO heuristic participants were (attitude: M_SEO_ = 4.27, SD_SEO_ = 0.69; acceptance: M_SEO_ = 3.58, SD_SEO_ = 1.09). Against this background, our baseline hypotheses (H0a and H0b) were clearly confirmed by the data.

**Table 2:** Differences in supportive attitude toward CCU and acceptance of CCU (pre-measurement) between SEO vs. SER heuristic participants.

Dependent Variable	Group	*N*	M (SD)	*F*	*p*-value	η_p_^2^
Supportive Attitude toward CCU	SEO	59	4.27 (0.69)	12.362	<0.001***	0.08
	SER	90	3.74 (1.02)			
Acceptance of CCU	SEO	59	3.58 (1.09)	23.672	<0.001***	0.14
	SER	90	2.59 (1.29)			

SEO = participants characterized by a strongly expressed optimization heuristic; SER = participants characterized by a strongly expressed restriction heuristic; data analyses were only carried with participants who provided complete data for all the variables examined. *p < 0.05; **p < 0.01; **p < 0.001.

### 3.2 Overall effect of a scenario-based intervention approach on SER heuristic participants’ supportive attitude and acceptance

To examine the overall effect of our scenario-based intervention approach on SER heuristic-participants’ attitude toward CCU and on their acceptance of CCU, we computed a repeated-measurement ANOVA for both dependent variables. In line with our research hypotheses H1a and H1b, the analyses identified a significant increase for SER heuristic-participants supportive attitude toward CCU [*F*_(1, 99)_ = 5.492, *p* < 0.01; η_p_^2^ = 0.05; M_SER-pre_ = 3.79, SD_SER-pre_ = 0.98; M_SER-post_ = 3.90, SD_SER-post_ = 0.95], as well as a highly significant increase of their acceptance of CCU [*F*_(1, 87)_ = 14.505, p < 0.001; η_p_^2^ = 0.14; M_SER-pre_ = 2.60, SD_SER-pre_ = 1.30; M_SER-post_ = 3.00, SD_SER-post_ = 1.34] from pre- to post-measurement (see Table 3 for an overview).

**Table 3:** Differences in attitude toward CCU and acceptance of CCU of SER heuristic participants between pre- and post-measurement.

Dependent Variable	Measurement	*N*	M (SD)	*F*	*p*-value	η_p_^2^
Attitude toward CCU	Pre	100	3.79 (0.98)	5.492	0.02*	0.05
	Post		3.90 (0.95)			
Acceptance of CCU	Pre	88	2.60 (1.30)	14.505	<0.001***	0.14
	Post		3.00 (1.34)			

Data analyses were only carried with participants who provided complete data for all the variables examined. *p < 0.05; **p < 0.01; ***p < 0.001.

### 3.3 Comparing effects in the dependent variables between baseline vignette-intervention vs. supplementary vignette-intervention

We examined possible differences in the effects of both types of vignette-interventions on SER heuristic participants attitude toward CCU and their acceptance of CCU by computing a repeated-measurement ANOVA for each dependent variable with vignette-intervention (baseline vs. supplementary) as the independent variable. Although data analyses identified significant main effects for both dependent variables (which is in line with the results presented in section 4.2), a significant interaction effect between time of measurement and vignette-intervention could not be identified, neither for SER heuristic participants’ attitude toward CCU [*F*_(1, 98)_ = 0.549, *p* = 0.46], nor for their acceptance of CCU [*F*_(1, 86)_ = 0.128, *p* = 0.72] (see Table 4 for all details). Thus, our research hypotheses H2a and H2b were not supported by the data.

**Table 4:** Differences in changes in attitude toward CCU and acceptance of CCU in SER heuristic participants depending on vignette-intervention.

Dependent Variable	Type of effect	Vignette-intervention	Measurement	*N*	M (SD)	*F*	*p*-value	η_p_^2^
Attitude toward CCU	Main effect	x_1_, x_2_	Pre	100	3.79 (0.98)	5.320	0.023*	0.05
			Post	100	3.90 (0.95)			
	Interaction effect	x_1_	Pre	48	3.87 (0.86)	0.549	0.46	--
			Post		3.94 (0.83)			
		x_2_	Pre	52	3.72 (1.07)			
			Post		3.87 (1.06)			
Acceptance of CCU	Main effect	x_1_, x_2_	Pre		2.59 (1.29)	14.479	<0.001***	0.14
			Post		3.01 (1.33)			
	Interaction effect	x_1_	Pre		2.41 (1.28)			
			Post		2.81 (1.24)			
		x_2_	Pre	47	2.76 (1.31)			
			Post		3.09 (1.43)			

x_1_ = basic vignette-intervention, x_2_ = supplementary vignette-intervention; data analyses were only carried with participants who provided complete data for all the variables examined. *p < 0.05; **p < 0.01; ***p < 0.001.

### 3.4 Examining vignette-intervention effects on SEO heuristic participants’ supportive attitude and acceptance

To finally examine possible (or the expected absent) overall effect of our scenario-based intervention approach on SEO heuristic-participants’ supportive attitude toward CCU and on their acceptance of CCU, we also computed a repeated-measurement ANOVA for both dependent variables in this specific group of participants. In contrast to the results of this analysis in the group of SER heuristic participants and, thus, in line with our expectations, no significant changes were identified in the analysis—neither for SEO heuristic participants’ supportive attitude toward CCU [*F*_(1, 64)_ = 3.296, *p* = 0.07], nor for their acceptance of CCU [*F*_(1, 58)_ = 2.221, *p* = 0.14; see Table 5 for all details]. Thus, our final research hypothesis H3 was confirmed by the data.

**Table 5:** Differences in supportive attitude toward CCU and acceptance of CCU of SEO heuristic participants between pre- and post-measurement.

Dependent Variable	Measurement	*N*	M (SD)	*F*	*p*-value	η_p_^2^
Supportive attitude toward CCU	Pre	65	4.27 (0.69)	3.296	0.07	--
	Post		4.34 (0.72)			
Acceptance of CCU	Pre	59	3.58 (1.09)	2.221	0.14	--
	Post		3.73 (1.24)			

Data analyses were only carried out with participants who provided complete data for all the variables examined. *p < 0.05; **p < 0.01; ***p < 0.001.

## 4 Discussion

The overall research aim of our study was to examine the effects of a strongly expressed restriction-oriented climate change mitigation heuristic (SER heuristic) on individuals’ supportive attitude toward and acceptance of negative emission technologies (CCMT) such as Carbon Capture and Utilization (CCU): In line with initial findings from previous research (Matthies et al., 2023) our study provides further empirical evidence for such effects by showing SER heuristic individuals reporting significantly lower supportive attitude toward CCU as well as significantly lower acceptance of CCU than individuals characterized by a strongly expressed optimization-oriented climate change mitigation heuristic (SEO heuristic individuals).

In addition to this examination of SER heuristic’s consequences on individuals’ attitude toward and acceptance of CCU, we further investigated the effects of a scenario-based intervention approach on individuals’ attitude and acceptance—especially referring to SER heuristic individuals: By doing so, we presented a scenario in which the combination of different climate protection strategies (i.e., sufficiency, efficiency and the use of CCMT like CCU) leads to the achievement of national climate protection goals.

The main function of our scenario-based intervention was to convey the necessity of sufficiency strategies in an integrative and thus highly effective national climate-protection program in an appreciative manner. The mistrust that technological strategies are used to avoid sufficiency measures should thus be prevented.

Our results imply our scenario-based intervention approach to be an effective tool for the promotion of SER heuristic individuals’ supportive attitude toward CCU as well as for their acceptance of CCU. In contrast to our expectations, we found no empirical evidence for stronger intervention effects, when presenting a slightly supplemented scenario-based intervention (supplemented by the additional appreciative emphasis on the importance of the sufficiency strategy).

Finally, our results imply the examined scenario-based intervention approach to be effective for the promotion of SER heuristic—individuals’ supportive attitude toward and acceptance of CCU, while no intervention effects were found for SEO heuristic individuals.

### 4.1 Implications for policymakers and practitioners working on climate change mitigation

From an application perspective, the lack of an intervention effect for the SEO heuristic individuals is positive: the scenario-based intervention (combination of sufficiency and efficiency strategies) apparently did not lead to reactance among the SEO heuristic individuals, making this communication strategy a feasible broad communication strategy.

The aim of this study was to test the effect of an intervention that aims to increase the acceptance of CCU^4^ for people with a strong restriction-oriented heuristic. In this study, the strong restriction-oriented heuristic was contrasted with an optimization heuristic, whereby these heuristics are not designed as opposites. These efforts are based on initial empirical findings that a very strong restriction heuristic can hinder the acceptance of innovative, highly effective technologies for mitigating climate change. The study at hand was able to show that providing easily accessible information about CCU technology in combination with a general support of the concept of sufficiency could lead to an increase in the supportive attitude toward and acceptance of CCU.

It is quite likely that the narrative of technical solutions triggers the concern among people with strong restriction heuristics that the (political) promotion of technical solutions goes hand in hand with an undermining of sufficiency strategies, i.e., that technical solutions are pursued in order to avoid sufficiency. If these urgently needed technologies to mitigate climate change are to be further supported, two key strategies must be pursued: firstly, transparent communication about the opportunities, risks and impact of the technologies, and secondly, communication that addresses the importance of sufficiency strategies in order to dispel concerns. Based on the results of this study, policymakers and/or practitioners could develop information campaigns using materials similar to those employed in this study. For example, online and/or social media campaigns could promote videos that explain the technologies in an easy-to-understand manner while credibly conveying the necessity of sufficiency strategies. Medium- and long-term credibility will depend on the extent to which accompanying sufficiency strategies are actually promoted and supported. Ideally, concrete examples of such approaches should be communicated.

Due to the urgency of the situation, all resources must be pooled in order to drive climate change mitigation forward. It can therefore no longer be a question of “either or.”

### 4.2 Limitations and further research

In the present paper, we were specifically interested in the role of restriction-oriented heuristics and optimization heuristics in the acceptance of CCMTs. We wanted to examine whether an intervention that combines information and communication about the necessity of sufficiency strategies can increase the acceptance of CCMTs among individuals with a strong restriction-oriented heuristic. In focusing on the experimental testing of this question, we deliberately accepted a very narrow consideration of the behavior (in this case, the acceptance of CCU). Having demonstrated the importance of these heuristics in the first step, the experimental vignette methodology (EVM) could be used in the future to systematically consider and examine the impact of interventions, as well as the interplay of the two heuristics of restriction versus optimization orientation with other variables in the Value belief Norm Model, such as personal norm, biocentric, anthropocentric, and egoistic values, competing beliefs, problem awareness, or even emotions that can influence problem awareness. The study by Matthies et al. (2023) has already shown positive correlations between biocentric values, personal norm, and the heuristics examined here. These analyses might also help identify possible conflicts between different heuristics and associated climate policy measures.

The sample was restricted to the German population. The findings presented and discussed here should therefore be considered embedded in their national context. In this context, it should be noted that the environmental movement in Germany in particular tends to be skeptical of technology, especially when it comes to large-scale plants. We therefore recommend that future studies also measure general affinity or skepticism toward technology. However, we assume that the relevance of the described heuristics and the impact of the tested intervention to transform a strong restriction heuristic into the direction of an optimization heuristic might be of relevance for other countries as well. Furthermore, the representativeness was further limited by the strict selection criteria in relation to the heuristics. However, the restriction of the sample was absolutely necessary in order to be able to realize the experimental design properly. At the same time, the strong sample reduction indicates that the proportion of people with very dominant heuristics in the population is probably not particularly large. However, as no reactance effects were found for the SEO heuristic participants, this does not represent a significant risk for the communication strategy proposed here.

Due to this strict selection criteria and the associated sample reduction, a simple experimental design was chosen for this study. Although the general effect of the intervention could be demonstrated by the pre-post-test, an additional control group would be recommended for future studies, which would allow the isolated effect of information provision to be tested without any reference to sufficiency strategies. With regards to the missing difference in the effects between both scenario-types (baseline vignette vs. supplementary vignette), the strict selection criteria and the associated sample reduction could also be of relevance: Considering the results of a power analysis carried out retrospectively, based on the remaining sample size usuable for comparing the effects in the dependent variables between both scenario-types (see Section 4.3 for details), our study provides sufficient power (ß = 0.80) with regards to at least medium-strength effects (*f* = 0.25), while the study’s power was insuffiencent for identifying small effects/differences between the scenario-types. Therefore, further research is needed to be able to investigate possible/missing differences in the effects of the scenarios more reliably by analysing data from bigger samples. Although it is unlikely to have had a negative impact on the central findings of our study, we would nevertheless like to point out a further limitation with regard to the measurement of the dependent variables. Since there are relevant conceptual as well as methodological imprecisions in the field of research on social acceptance of sustainable and renewable energy technologies (see again, e.g., Bonaiuto et al., 2024 or Milani et al., 2024 for an overview), it should be mentioned, that such imprecisions are partially also represented in our measurement of participants’ supportive attitude toward CCU and their acceptance of CCU: As described in Section 3.2, we decided on the final item composition for measuring these two constructs on the basis of a factor analysis conducted with the captured data. Although this factor analysis clearly supports the assumption of two, albeit significantly correlated, factors, from a conceptually point of view the following issue should at least be pointed out: Items referring to statements of “support for CCU,” which were integrated into the attitude-measure in the present study, seem to (also) refer to a behavioral response toward CCU and, thus, such items could (also) represent an acceptance-measure for CCU. Even if, as already mentioned, this issue should probably not have had a problematic influence on the results of the present study, having in mind the nessecary increasing importance of differentiating attitudes, intentions, and behaviors in environmental psychology, future research should consider this issue in developing appropraite measures for capturing dependent variables in the context of social acceptance of sustainable and renewable energy technologies.

In our view, the present findings already provide helpful indications for future target-oriented communication of necessary CCMTs. For future studies, it would also be interesting to investigate in more detail how widespread the various heuristics actually are and to what extent they are connected to different stakeholder groups. However, even if the findings confirm the use of the underlying heuristics, it seems promising to analyze the structures of these heuristics even more in-depth, especially with regard to conflict potentials.

## Data Availability

The raw data supporting the conclusions of this article will be made available by the authors, without undue reservation.

## Footnotes

^1^Please note: The terms used to describe the groups do not completely correspond with the pre-registration terms. The content is consistent.

^2^Within the inclusion criterion procedure, we have further used a stratification-procedure resulting in a 50:50% ratio between participants, who chose the sufficiency- vs. the efficiency-measure.

^3^Difference-value for each participant from both scales (restriction heuristic – optimization heuristic), representing a holistic measure for participants’ expressed restriction heuristic.

^4^The CCU technology was selected as a substitute for innovative technologies to mitigate climate change.
