# Supplementary material for: Overcoming heuristics that hinder people’s acceptance of climate-change-mitigation technologies
Source: Front Psychol. 2025 Jun 18;16:1433280. doi: 10.3389/fpsyg.2025.1433280 (PMC12213510; doi:10.3389/fpsyg.2025.1433280)
Supplement: Supplementary file 1 [file Supplementary_file_1.docx]

**Appendix**

**Table A.1.**

Scales/ items used in the study and descriptive statistics.

| **Scale/ items** | **Number of items** | **Formulation** | **Reply options** | **Reliability** | **M (SD)** | **Min** | **Max** |
| --- | --- | --- | --- | --- | --- | --- | --- |
| Attitudes toward CCU | 6 | What is your attitude towards Carbon Capture and Utilization (CCU; CO2 capture and subsequent use of carbon, e.g. bioplastics as a building material) as a technology to limit climate change?   - referring to CCU in general - referring to the use of CCU for the medium-term storage of emissions (from the production of plastics) - referring to the use of CCU and for the long-term storage of emissions (from the production of building materials)   How much do you agree with the following statements about Carbon Capture and Utilization (CCU; CO2 capture and subsequent use of carbon, e.g. bioplastics as a building material) as a technology to limit climate change? I support Carbon Capture and Utilization."   - referring to CCU in general - referring to the use of CCU for the medium-term storage of emissions (from the production of plastics) - referring to the use of CCU and for the long-term storage of emissions (from the production of building materials) | (1) “very much against it” - (5) “very much in favor”  (1) “strongly disagree” - (5) “strongly agree” | α = .92 (pre)  α = .92 (post) | pre: 3.98 (0.90)  post: 4.09 (0.89) | 1 | 5 |
| Acceptance of CCU | 3 | How much do you agree with the following statements about Carbon Capture and Utilization (CCU; CO2 capture and subsequent use of carbon, e.g. bioplastics as a building material) as a technology to limit climate change? I try to convince others of the importance of Carbon Capture and Utilization.   - referring to CCU in general - referring to the use of CCU for the medium-term storage of emissions (from the production of plastics) - referring to the use of CCU and for the long-term storage of emissions (from the production of building materials) | (1) “strongly disagree” - (5) “strongly agree” | α = .93 (pre)  α = .92 (post) | pre: 2.96 (1.32)  post: 3.29 (1.34) |  |  |
| Expressed Restriction Heuristic | 5 | How much do you agree with the following statement?   - We have asked far too much of our planet in recent years, so now we have to pay the price and do without. - We cannot buy our way out of the climate crisis. - We in the western industrialized nations are to blame for the climate crisis and must now bear the consequences. - Restrictions on climate protection are the just punishment for our overconsumption. - The Western lifestyle is the cause of climate change, and it is only fair that we are now affected by severe restrictions. | (1) “strongly disagree” - (5) “strongly agree” | α = .94 | 3.48 (1.19) |  |  |
| Expressed Optimization Heuristic | 4 | How much do you agree with the following statement?   - As citizens of an industrialized nation, we can contribute to solving the global climate crisis primarily through investment. - In order to limit the climate crisis quickly, each individual should implement measures in their own area that save a particularly large amount of CO_2_. - I am prepared to invest money to limit the climate crisis. - Everyone should know their carbon footprint so that they can start where it makes the most difference. | (1) “strongly disagree” - (5) “strongly agree” | α = .73 | 3.22 (0.96) |  |  |
| Attention check | 1 | Please briefly answer the following question about the Carbon Capture and Utilization (CCU):  In which products can **NO** CO_2_ be stored using CCU? | In food; In fuels;  In building materials; In plastics; I do not know. ^(a)^ | -- | -- | -- | -- |

Note: M = mean value; SD = standard deviation; Min = Minimum; Max = Maximum. ^(a)^ participants, who gave a wrong answer (“in food” or “I do not know”) to this attention check question were excluded.

**Table A.2.**

Factor loadings of all items, calculated in exploratory factor analyses (extraction method: Principal Axis Factoring; rotation method: Oblimin, Delta = 0) conducted with pretest data of the dependent variables (N = 144).

| **Items** | **Loadings on factor 1 (supportive Atttitudes towards CCU)** | **Loadings on factor 2 (acceptance of CCU)** |
| --- | --- | --- |
| What is your attitude towards Carbon Capture and Utilization (CCU; CO2 capture and subsequent use of carbon, e.g. bioplastics as a building material) as a technology to limit climate change?   - referring to CCU in general - referring to the use of CCU for the medium-term storage of emissions (from the production of plastics) - referring to the use of CCU and for the long-term storage of emissions (from the production of building materials)   How much do you agree with the following statements about Carbon Capture and Utilization (CCU; CO2 capture and subsequent use of carbon, e.g. bioplastics as a building material) as a technology to limit climate change? I support Carbon Capture and Utilization."   - referring to CCU in general - referring to the use of CCU for the medium-term storage of emissions (from the production of plastics) - referring to the use of CCU and for the long-term storage of emissions (from the production of building materials) | .86  .72  .82  .91  .77  .90 | .00  .05  -.01  .-.01  .01  -.03 |
| How much do you agree with the following statements about Carbon Capture and Utilization (CCU; CO2 capture and subsequent use of carbon, e.g. bioplastics as a building material) as a technology to limit climate change? I try to convince others of the importance of Carbon Capture and Utilization.   - referring to CCU in general - referring to the use of CCU for the medium-term storage of emissions (from the production of plastics) - referring to the use of CCU and for the long-term storage of emissions (from the production of building materials) | .04  .04  -.06 | .87  .87  .94 |

Note: Explained variance = 73.05%; correlation between both factors: r = .54 (p<.001).
