# Supplementary material for: Overcoming heuristics that hinder people’s acceptance of climate-change-mitigation technologies
Source: Front Psychol. 2025 Jun 18;16:1433280. doi: 10.3389/fpsyg.2025.1433280 (PMC12213510; doi:10.3389/fpsyg.2025.1433280)
Supplement: Supplementary file 2 [file Supplementary_file_2.docx]

**Appendix B**

**B.1: Script used for the video short video providing general information about CCU:**

*In order to achieve the Paris climate targets and the associated 1.5-degree target, emissions on earth need to be drastically reduced. Our current greenhouse gas emissions take on proportions that we simply cannot compensate for in the long run.*

*To counteract this, carbon must be extracted from the atmosphere in the short and long term. One technology that temporarily stores CO_2_ in other products is CCU: carbon capture and utilization. With this technology, CO_2_ is either extracted directly from the air or from combustion exhaust gasses, stored, and subsequently transformed into one of many possible products. The wide range of possible products spans from base chemicals such as methanol, to fuels, plastics (for example mattresses or car interiors) and construction materials. The longer the lifespan of the product, the better its climate balance: a long product lifespan ensures that carbon is kept out of the atmosphere for a longer period of time. Fuels produced with CCU therefore only have a very limited carbon storage effect, as they quickly return CO_2_ to the atmosphere. Plastics can store carbon for around 10 to 20 years, depending on the product. Construction materials can withdraw and store carbon from the atmosphere for up to 100 years.*

**B.2: Script used for the video-based vignette 1 (baseline vignette):**

*The year is 2060. As planned, Germany’s national climate protection goals were successfully implemented through various climate protection strategies in politics, industry, and society. Citizens increasingly adjusted their consumption patterns regarding diet, mobility, and all other areas of daily life to achieve more sufficient, climate-friendly consumption behaviors. At the same time, the CO_2_ emissions of all industrial processes and technologies were increasingly optimized and the remaining climate-damaging emissions compensated through the use of negative emissions technologies such as CCU.*

*Thereby, CCU is predominantly used for the production of long-lasting carbon-storage products such as construction materials. Short-term carbon-storage products such as fuels are rarely produced using CCU, and only to guarantee a safety net of necessary infrastructure like emergency power generators for hospitals.*

**B.3: Script used for the video-based vignette 2 (supplementary vignette):**

*The year is 2060. As planned, Germany’s national climate protection goals were successfully implemented through various climate protection strategies in politics, industry, and society. Citizens increasingly adjusted their consumption patterns regarding diet, mobility, and all other areas of daily life to achieve more sufficient, climate-friendly consumption behaviors. At the same time, the CO_2_ emissions of all industrial processes and technologies were increasingly optimized and the remaining climate-damaging emissions compensated through the use of negative emissions technologies such as CCU.*

*Thereby, CCU is predominantly used for the production of long-lasting carbon-storage products such as construction materials. Short-term carbon-storage products such as fuels are rarely produced with CCU, and only to guarantee a safety net of necessary infrastructure like emergency power generators for hospitals.*

*Politics, industry and society agree, that the climate protection goals were only successfully met due to the combination of these different strategies. The promotion of sufficient lifestyles continues to be regarded as the most important factor for climate protection.*
